# The short-chain fatty acid propionate prevents ox-LDL-induced coronary microvascular dysfunction by alleviating endoplasmic reticulum stress in HCMECs

**DOI:** 10.1371/journal.pone.0304551

**Published:** 2024-05-30

**Authors:** Dan Hong, Wen Tang, Fei Li, Yating Liu, Xiao Fu, Qin Xu

**Affiliations:** 1 Department of Geriatric Medicine, Xiangya Hospital, Central South University, Hunan, China; 2 Department of Hematology Medicine, Xiangya Hospital, Central South University, Hunan, China; 3 Department of Cardiology Medicine, Brain Hospital of Hunan Province, Hunan, China; Georgia State University, UNITED STATES

## Abstract

Coronary microvascular dysfunction (CMD) is a critical pathogenesis of cardiovascular diseases. Lower endothelial nitric oxide synthase (eNOS) phosphorylation leads to reduced endothelium-derived relaxing factor nitric oxide (NO) generation, causing and accelerating CMD. Endoplasmic reticulum stress (ER stress) has been shown to reduce NO production in umbilical vein endothelial cells. Oxidized low-density lipoprotein (ox-LDL) damages endothelial cell function. However, the relationship between ox-LDL and coronary microcirculation has yet to be assessed. Short-chain fatty acid (SCFA), a fermentation product of the gut microbiome, could improve endothelial-dependent vasodilation in human adipose arterioles, but the effect of SCFA on coronary microcirculation is unclear. In this study, we found ox-LDL stimulated expression of ER chaperone GRP78. Further, we activated downstream PERK/eIF2a, IRE1/JNK, and ATF6 signaling pathways, decreasing eNOS phosphorylation and NO production in human cardiac microvascular endothelial. Furthermore, SCFA-propionate can inhibit ox-LDL-induced eNOS phosphorylation reduction and raise NO production; the mechanism is related to the inhibition of ER stress and downstream signaling pathways PERK/eIF2a, IRE1/JNK, and ATF6. In summary, we demonstrate that ox-LDL induced CMD by activating ER stress, propionate can effectively counteract the adverse effects of ox-LDL and protect coronary microcirculation function via inhibiting ER stress.

## Introduction

Coronary microvascular dysfunction (CMD) is a structural or functional disorder in coronary microvessels with an inner diameter of less than 400μ [1]. CMD is an essential pathogenesis of cardiovascular diseases such as acute coronary syndrome, stable angina pectoris, X syndrome, hypertrophic cardiomyopathy, diabetic cardiomyopathy, and no-reflow phenomenon after stent implantation [2]. The fundamental mechanism of CMD is endothelial-dependent or endothelial-independent coronary microvascular vasomotor function dysregulation. Endothelial-dependent dysregulation is caused by an imbalance of vascular dilators derived from endothelial cells, such as the reduction of vasodilator factor nitric oxide (NO) or increased constrictors. Studies have shown that endoplasmic reticulum stress (ER stress) plays a crucial role in inducing endothelial dysfunction and atherosclerosis [3], which can reduce NO production in umbilical vein endothelial cells [4]. ER stress may become a new therapeutic target for cardiovascular diseases [5].

The gut microbiome has been identified as the ninth system of the human body, and studies have shown that supplementing probiotics can improve liver sinusoidal endothelial dysfunction in rats [6] and significantly improve endothelial-dependent vasodilation in human adipose arterioles, which is associated with an increase in NO bioavailability [7]. Dietary fiber produces short-chain fatty acids (SCFA) after fermentation by the gut microbiome, including propionate, butyrate, and acetate. Propionate significantly attenuated vascular dysfunction and decreased aortic atherosclerotic lesion area in mice [8]. Currently, there is rare research on the relationship between gut microbiota and coronary microvascular function, and whether SCFA can improve coronary microvascular endothelial cell function by inhibiting ER stress remains to be explored.

Dyslipidemia is one of the critical risk factors for obstructive coronary artery disease. Oxidized low-density lipoprotein (ox-LDL) damages endothelial cell function and promotes the progression of atherosclerosis. Therefore, lipid-lowering therapy is one of the core components of treating coronary heart disease. Coronary microvascular endothelial dysfunction occurs earlier than epicardial endothelial dysfunction [9], and the influence of dyslipidemia on coronary microcirculation function is currently controversial. Clinical studies have found that lowering LDL levels can quickly improve coronary microvascular function [10]. However, some studies report that hyperglycemia significantly impacts coronary microvascular function more than dyslipidemia [11]. Basic research on the relationship between LDL and coronary microcirculation still needs to be completed, and its potential molecular mechanisms still need clarification. Whether standard lipid-lowering therapy needs to be initiated in advance requires further exploration.

In summary, this study aims to clarify the effect of ox-LDL on coronary microvascular endothelial cell function, explore whether ER stress is involved in the process, and investigate whether the SCFA-propionate produced by gut microbiome metabolism can alleviate ER stress and improve the damage of ox-LDL to coronary microvascular endothelial cell function. This research will provide more evidence for the timing of initiating lipid-lowering therapy and the treatment of coronary microcirculation dysfunction in clinical practice.

## Results

### Impact of ox-LDL on ER stress, endothelial nitric oxide synthase (eNOS) phosphorylation, and NO release in Human Cardiac Microvascular Endothelial Cells (HCMECs)

**Fig 1:**
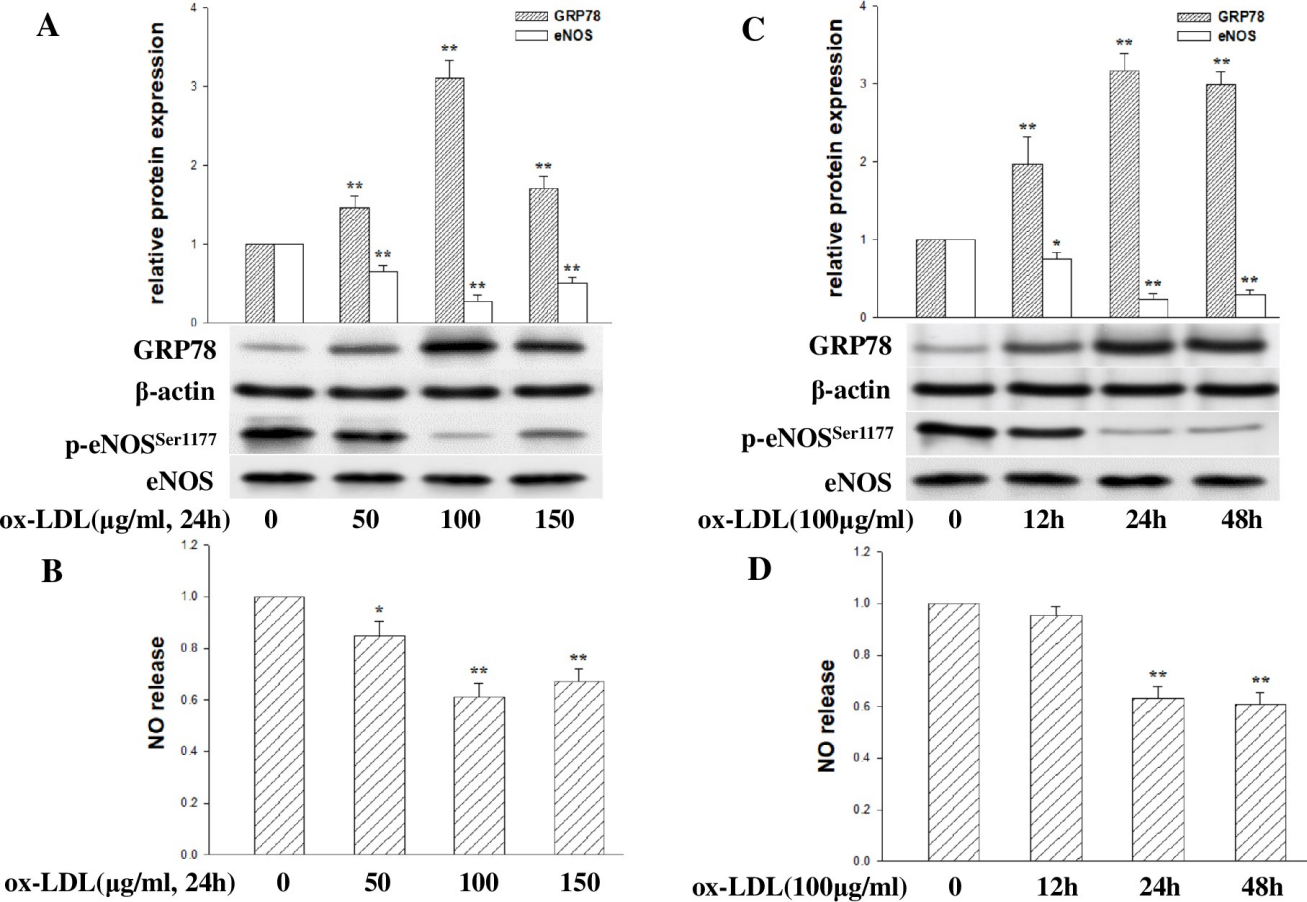
ox-LDL triggers ER stress activation and impairs HCMECs function. (A) The protein levels of GRP78, eNOS(Ser1177) phosphorylation and total eNOS were detected by Western blot after ox-LDL treatment at various concentrations (0, 50, 100, or 150 μg/ml) for 24 hours. (B) Griess reaction observed nitric oxide (NO) production after ox-LDL treatment (0–150 μg/ml) for 24 hours. (C) The protein levels of GRP78, eNOS(Ser1177) phosphorylation, and total eNOS were detected after exposure to 100 μg/ml ox-LDL at various times (0, 12, 24, or 48 hours). (D) NO production after ox-LDL treatment (0–150 μg/ml) for 24 hours. The data were expressed as the mean ± SD, n = 3. Compared with control, *P<0.05; **P <0.01.

To understand the impact of ox-LDL on ER stress and eNOS phosphorylation in HCMECs, cells were treated with varying concentrations of ox-LDL (50–150 μg/ml) for 24 hours. Western blot analysis showed that treatment with ox-LDL upregulated the expression of ER chaperone GRP78 in a dose manner while downregulating eNOS(Ser1177) phosphorylation at doses of 50–100 μg/ml. High-concentration ox-LDL (150 μg/ml) did not show a more substantial induction effect (Fig 1A). Griess analysis confirmed that NO release downregulation mediated by ox-LDL treatment after incubation at the dose of 50–150 μg/mL, peaked at 100 μg/ml (Fig 1B). Furthermore, incubation with 100 μg/ml ox-LDL for 12–24 hours resulted in a time-dependent increase in GRP78 expression and a decrease in eNOS(Ser1177) phosphorylation. After 48 hours of incubation, the effect on GRP78 and p-eNOS(Ser1177) expression was weaker (Fig 1C). Additionally, NO release downregulation mediated by ox-LDL treatment after incubation for 24–48 hours, 12 hours of ox-LDL treatment does not affect NO release (Fig 1D). These findings suggest that ER stress triggered by ox-LDL may inhibit eNOS phosphorylation and reduce the NO release of HCMECs.

### Involvement of the Unfolded Protein Response (UPR) in ox-LDL-induced eNOS phosphorylation and NO release in HCMECs

**Fig 2:**
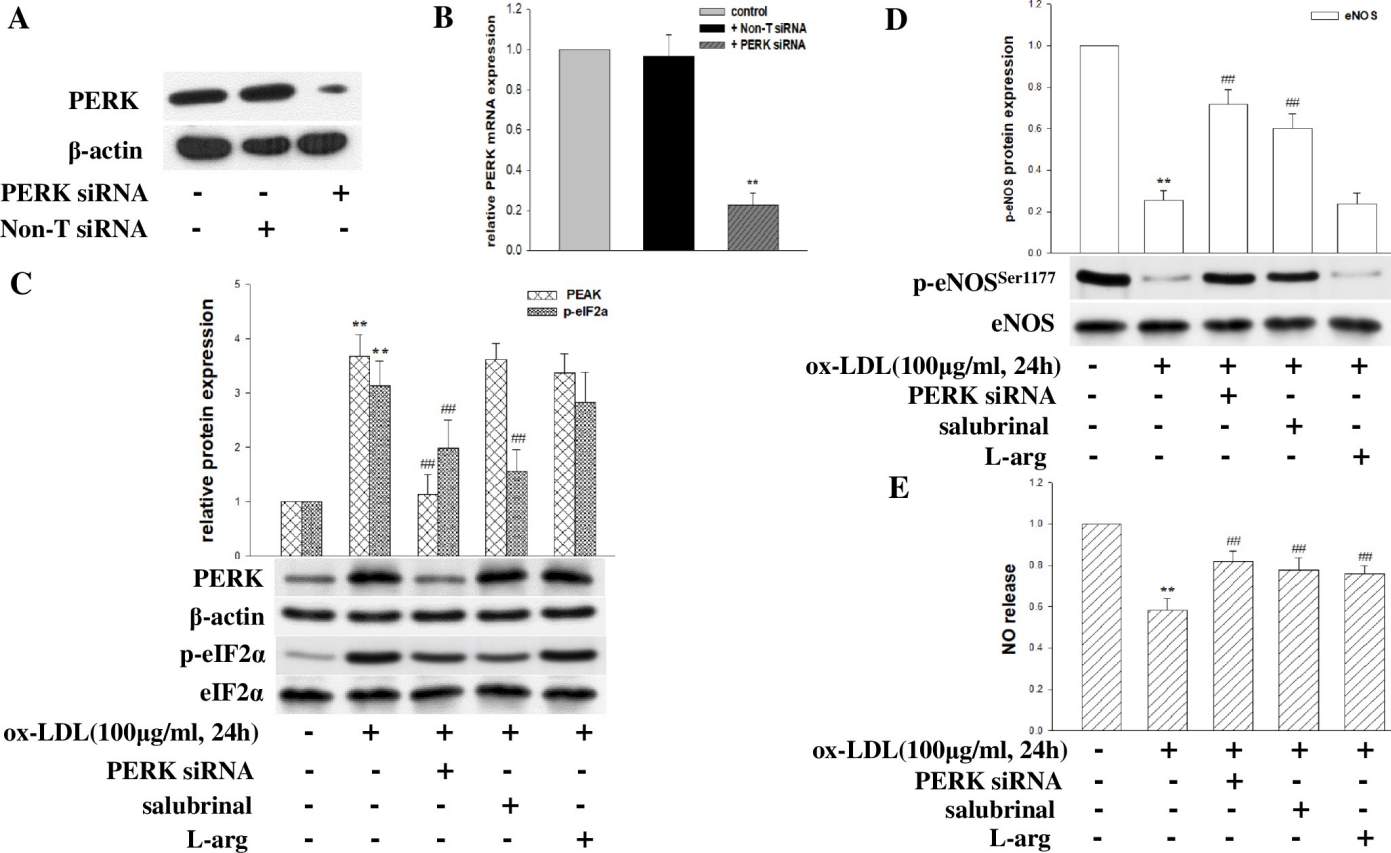
The role of the ER stress sensor PERK in ox-LDL-induced NO decrease in HCMECs. (A-B) PERK siRNA effectively suppressed the mRNA and protein expression of PERK. HCMECs were transfected with PERK siRNA, pretreated with inhibitor or L-arginine for 6 hours, and then exposed to ox-LDL (100μg/ml, 24 hours). (C) The protein levels of PERK and its downstream effector eIF2α phosphorylation were detected by Western blot. (D) eNOS phosphorylation and total eNOS were detected by Western blot. (E) NO production was detected by Griess reaction. The data were expressed as the mean ± SD, n = 3. Compared with the control, *P<0.05; **P<0.01. Compared with ox-LDL, #P<0.05; ##P<0.01.

**Fig 3:**
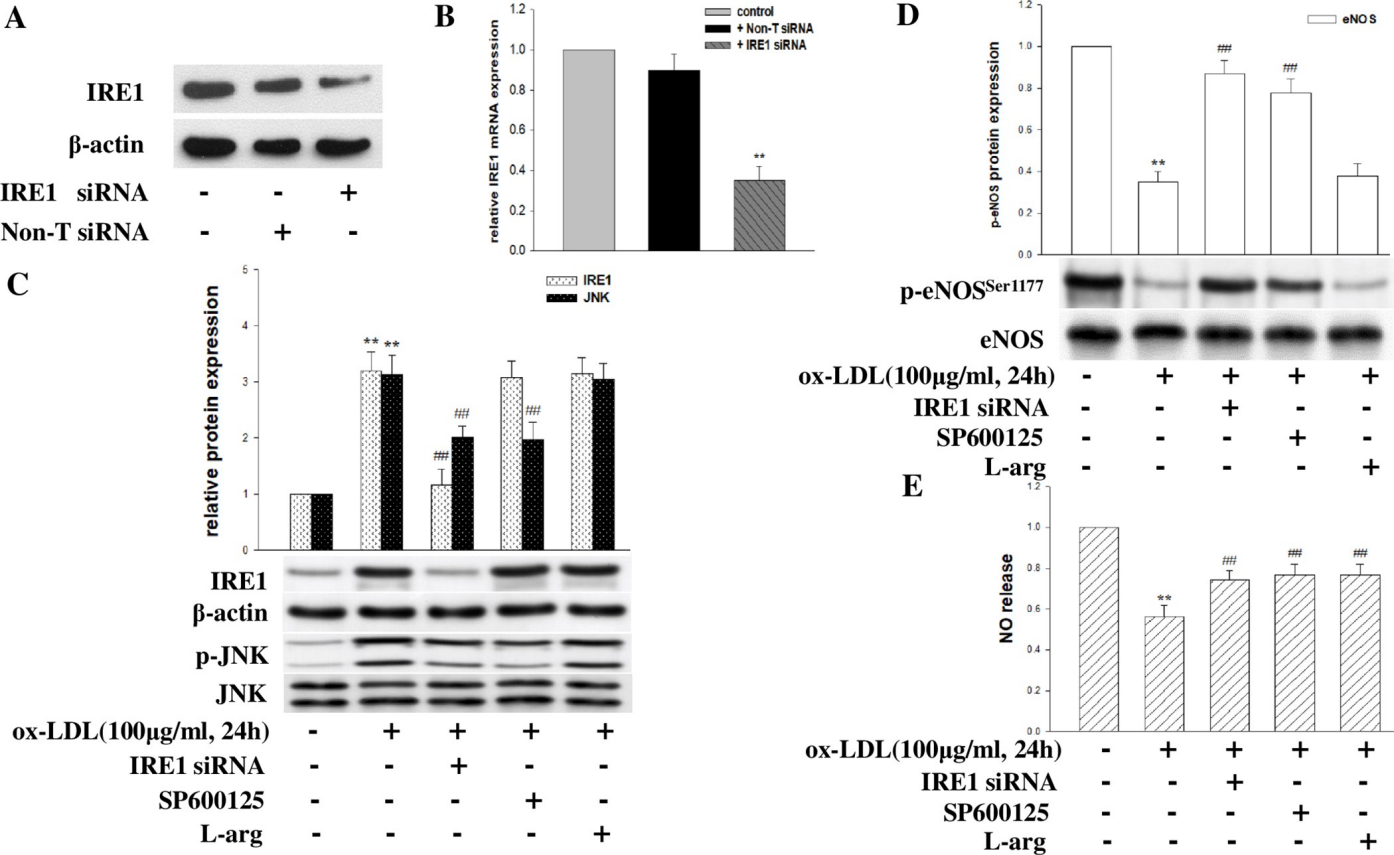
The role of the ER stress sensor IRE1 in ox-LDL-induced NO decrease in HCMECs. (A-B) IRE1 siRNA effectively suppressed the mRNA and protein expression of IRE1. HCMECs were transfected with IRE1 siRNA, pretreated with inhibitor or L-arginine for 6 hours, and then exposed to ox-LDL (100μg/ml, 24 hours). (C) The protein levels of IRE1 and its downstream effector JNK phosphorylation were detected by Western blot. (D) eNOS phosphorylation and total eNOS were detected by Western blot. (E) NO production was detected by Griess reaction. The data were expressed as the mean ± SD, n = 3. Compared with the control, *P<0.05; **P<0.01. Compared with ox-LDL, #P<0.05; ##P<0.01.

**Fig 4:**
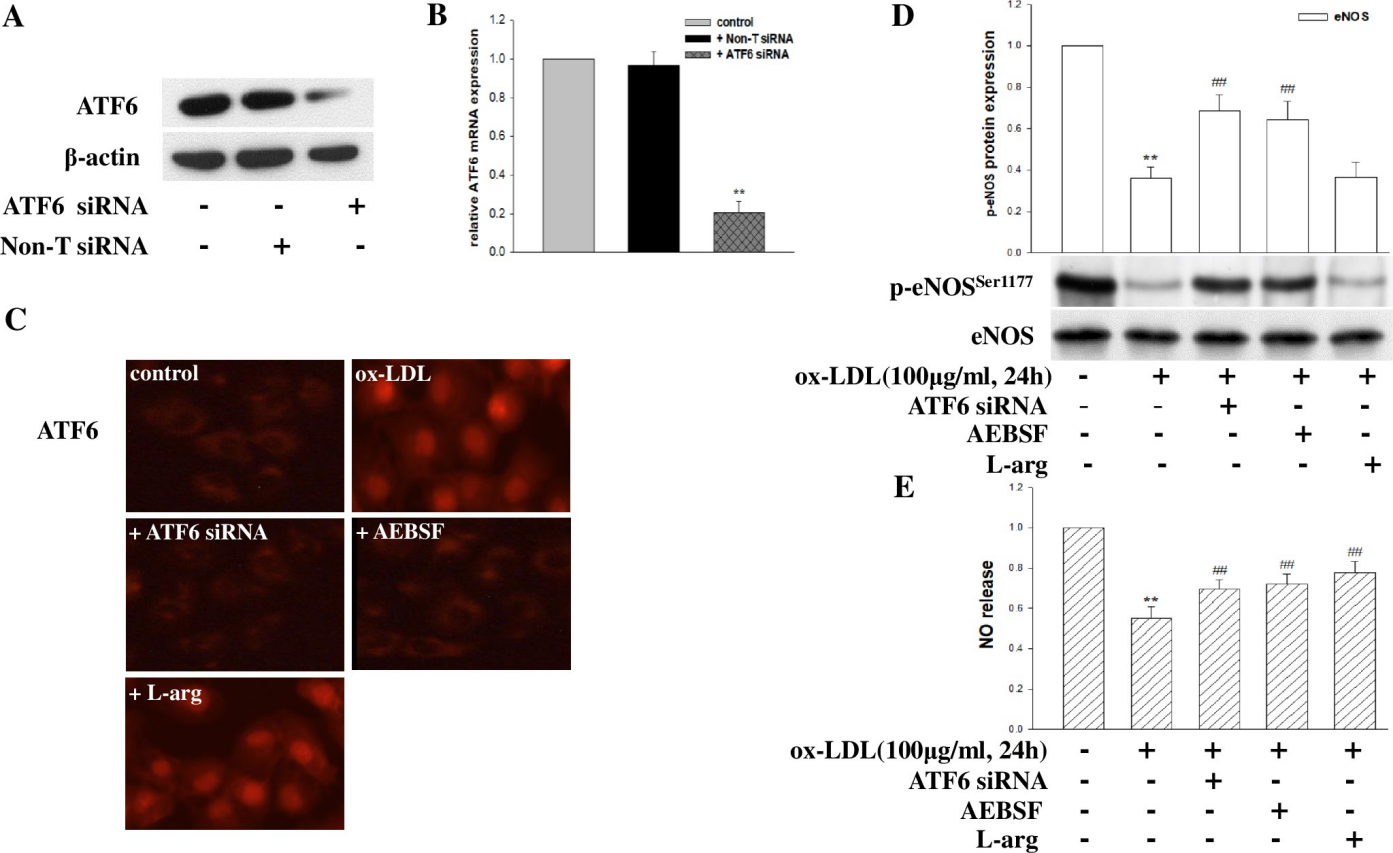
The role of the ER stress sensor ATF6 in ox-LDL-induced NO decrease in HCMECs. (A-B) siRNA effectively suppressed the mRNA and protein expression of ATF6. HCMECs were transfected with ATF6 siRNA, pretreated with inhibitor or L-arginine for 6 hours, and then exposed to ox-LDL (100μg/ml, 24 hours). (C) Immunofluorescence detected the protein expression of ATF6 (red) in HCMECs. (D) eNOS phosphorylation and total eNOS were detected by Western blot. (E) NO production was detected by Griess reaction. The data were expressed as the mean ± SD, n = 3. Compared with the control, *P<0.05; **P<0.01. Compared with ox-LDL, #P<0.05; ##P<0.01.

ER stress is characterized by the activation of ER stress sensors such as PERK, IRE1, and ATF6, leading to an unfolded protein response (UPR). To investigate the role of UPR in mediating ox-LDL-induced eNOS(Ser1177) phosphorylation and NO release decrease, HCMECs were transfected with siRNA or specific inhibitors for 6 hours, then exposed to ox-LDL (100 μg/ml) for 24 hours. As expected, treatment with siRNAs targeting PERK (Fig 2A and 2B), IRE1 (Fig 3A and 3B), and ATF6 (Fig 4A and 4B) reduced the target protein levels and mRNA expression. Furthermore, ox-LDL treatment increased PERK and its downstream target phospho-eIF2a expression, and pretreatment with PERK siRNA and eIF2a-specific inhibitor salubrinal inhibited the PERK/eIF2a pathway induced by ox-LDL (Fig 2C), as well as increased the levels of eNOS(Ser1177) phosphorylation and NO release (Fig 2D, 2E). Similarly, IRE1 siRNA and JNK inhibitor SP600125 inhibited the IRE1/JNK pathway induced by ox-LDL (Fig 3C), alleviating LDL-induced eNOS(Ser1177) phosphorylation downregulation and NO production decrease (Fig 3D and 3E). Additionally, pretreatment of HCMECs with ATF6 siRNA or inhibitor AEBSF before ox-LDL treatment inhibited the ATF6 activate induced by ox-LDL (Fig 4C), in addition, increased eNOS(Ser1177) phosphorylation and NO production (Fig 4D and 4E). However, non-targeting siRNA had no effect, and NO substrate L-arginine only affected the level of NO production. These results confirm the critical role of ox-LDL in regulating the ER stress pathway in generating NO in HCMECs.

### Effects of Short-Chain Fatty Acid (SCFA) propionate on ox-LDL-mediated ER stress and downregulation of eNOS phosphorylation and NO release

**Fig 5:**
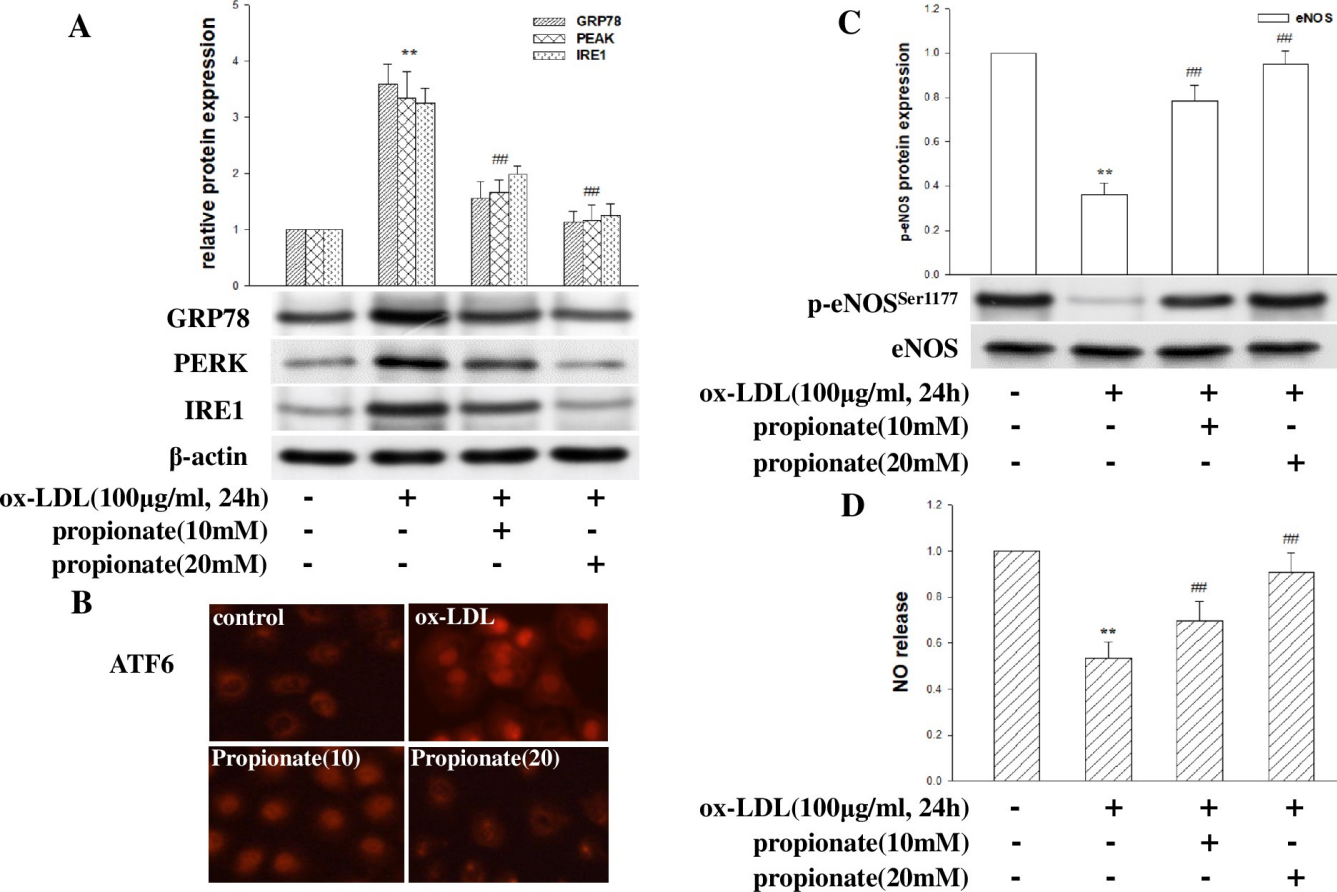
The impact of propionate on ox-LDL-induced ER stress and NO release in HCMECs. HCMECs were treated with different concentrations (10 and 20 mM) of propionate for 2 hours and then exposed to ox-LDL (100μg/ml, 24hours). (A) The protein levels of GRP78, PERK, and IRE1 were detected by Western blot. (B) Immunofluorescence detected the activation of ATF6. (C) eNOS phosphorylation and total eNOS were detected by Western blot. (D) NO production was detected by Griess reaction. The data were expressed as the mean ± SD, n = 3. Compared with the control, *P<0.05; **P<0.01. Compared with ox-LDL, #P<0.05; ##P<0.01.

As SCFA protects the cardiovascular system, ER stress may regulate the ox-LDL-mediated downregulation of p-eNOS(Ser1177) expression in HCMECs. Therefore, we investigated the potential effect of propionate on the ox-LDL-mediated activation of ER stress and downregulation of NO. As shown in Fig 5A and 5B, incubation with propionate (10–20 mM) inhibited the expression of GRP78 induced by ox-LDL and reduced the expression levels of PERK, IRE1, and ATF6 in HCMECs. Moreover, propionate increased eNOS(Ser1177) phosphorylation and NO release (Fig 5C and 5D). The most potent protective effect of propionate was observed in the presence of 20 mM in HCMECs. These data suggest that the protective effect of propionate on the ox-LDL-induced NO decrease in HCMECs could be mediated through the ER stress signaling pathway.

## Discussion

In this study, we found that: 1) ox-LDL reduces eNOS phosphorylation and decreases NO production in HCMECs in a dose- and time-dependent manner; 2) this process involves the initiation of ER stress, as activating PERK/eIF2α, IRE1/JNK1, and ATF6 signaling pathway; 3) Propionate protected the phospho-eNOS expression and NO level in ox-LDL-treated HCMECs; 4) Propionate may function via suppressing the ER stress.

Dyslipidemia is a core risk factor for the occurrence and development of atherosclerotic cardiovascular disease (ASCVD), and reducing LDL levels can effectively reduce the incidence of ASCVD and major adverse cardiovascular events (MACE) [12]. Existing studies have confirmed that CMD is a vital pathogenesis of ASCVD. Microcirculatory endothelial dysfunction precedes epicardial coronary endothelial dysfunction [9], and early microcirculatory dysfunction will accelerate the process of epicardial coronary atherosclerosis. Positive interventions for CMD may be one approach to preventing and treating ASCVD. Many studies have confirmed that LDL is a core risk factor for ASCVD. However, the effect of ox-LDL on coronary microcirculation function still needs more research to be elucidated, and whether early initiation of lipid-lowering therapy can prevent CMD and slow down the occurrence and development of ASCVD needs to be explored.

The main features of CMD include vascular endothelial injury, microvascular rarefaction, capillary vessel spasm, microvascular embolism, etc [13]. Multiple pathological factors damage the structure and function of cardiac microvascular endothelial cells (CMECs) that affect the concentration and expression of phosphorylation subtypes of eNOS in endothelial cells, reducing NO concentration [14–16]. NO is catalyzed by substrate L-arginine and is a critical factor in sustaining vasodilation. NO, and KATP regulate microvascular vasomotor due to no innervation [17]. When CMECs are impaired, with a reduction in NO concentration, a disruption in endothelial-dependent vasodilation eventually leads to coronary microcirculation dysfunction. Previous clinical studies have found that reducing LDL levels can rapidly improve coronary microcirculation function [10]. In our study, we found that ox-LDL could reduce eNOS phosphorylation in HCMECs, leading to a decrease in NO production and resulting in impairing the function of CMECs, which indicates the timing of clinical initiation of lipid-regulating needs to be advanced. In addition, we observed undesirable morphological changes and increased mortality of cells treated with ox-LDL at high concentrations (150 μg/ml) or for a long time (48 hours). So, we used flow cytometry analysis to detect the apoptosis of HCMECs. As shown in S1A and S1B Fig, the apoptosis of 50–100 μg/ml or 12–24 hours ox-LDL group was not increased compared with the control group. The apoptosis was increased in the groups treated with 150 μg/ml and 48 hours ox-LDL. It is speculated that high levels of LDL can activate other pathways besides ER stress, such as calcium overload and activation of pro-apoptotic factors, the underlying mechanism needs to be explored.

ER stress, induced by the accumulation of misfolded proteins in the ER, initiates the unfolded protein response (UPR), including IRE1, PERK, and ATF6 pathways. Previous studies have found that ER stress is involved in peripheral microvascular endothelial insufficiency in models of hyperlipidemia and diabetes mellitus [18, 19]. The previous research of our group confirmed that ox-LDL promoted apoptosis of umbilical vein endothelial cells by activating ER stress [20]. This study demonstrated that ox-LDL stimulated the expression of ER chaperone GRP78 and further activated downstream PERK/eIF2a, IRE1/JNK, and ATF6 signaling pathways by using siRNA and UPR-specific inhibitor, resulting in a decrease in eNOS phosphorylation and NO production in HCMECs. We validated that ER stress is involved in ox-LDL impairing CMEC function and may be one of the regulatory mechanisms of coronary microcirculation function and a potential new therapeutic target.

The gut microbiota is an essential component of human physiology and metabolic homeostasis. Existing research has found that the gut microbiota is closely related to blood pressure regulation [21], glucose and lipid metabolism, and the process of atherosclerosis [22]. *Bifidobacteria* significantly improve the endothelium-dependent vascular relaxation function of human adipose arterioles, related to increased NO bioavailability [9]. The influence of gut microbiota is mainly achieved through its metabolic products, such as short-chain fatty acid (SCFA) [23]. SCFA is mainly produced by bacterial fermentation of dietary fiber in the colon [24]. Propionate is a type of SCFA that can lower blood pressure in mice and reduce the risk of cardiovascular diseases [25–27]. This study first explored the protective effect of propionate on HCMEC function and confirmed that propionate can effectively protect against ox-LDL-induced HCMEC functional damage. It can inhibit ox-LDL-induced eNOS phosphorylation reduction and raise NO production, not causing apoptosis (S1C Fig); the mechanism may be related to the inhibition of ER stress and downstream signaling pathways PERK/eIF2a, IRE1/JNK, and ATF6. This study suggests that propionate can counteract the adverse effects of ox-LDL and protect coronary microcirculation function.

We demonstrated that ox-LDL impairs HCMEC function by inducing ER stress, but the signaling pathway is unknown. Inflammatory and oxidative stress are key in the pathogenesis of coronary microcirculation dysfunction [28]. Inflammatory cytokines enhance reactive oxygen species (ROS) production, decreasing NO availability and impairing vasodilation [29, 30]. Consistent with previous research [31, 32], we observed that the level of inflammatory cytokines, such as interleukin (IL)-1β, IL-6, and tumor necrosis factor (TNF)-α in HCMECs were increased upon ox-LDL treatment, as shown in S2A and S2B Fig. In terms of oxidative stress, ox-LDL treatment triggered the increase in ROS (S2C Fig). We speculate that inflammatory factors and oxidative stress may mediate the ER stress in endothelial cells of coronary microcirculation, and more molecular signals need further clarification. In addition, propionate can effectively reduce the IL-6, TNF-α, and ROS in ox-LDL-treated HCMECs (S2 Fig), demonstrating propionate reduces cytotoxicity of ox-LDL. Interestingly, propionate cannot inhibit the increase of IL-1β induced by ox-LDL.

## Conclusion

**Fig 6:**
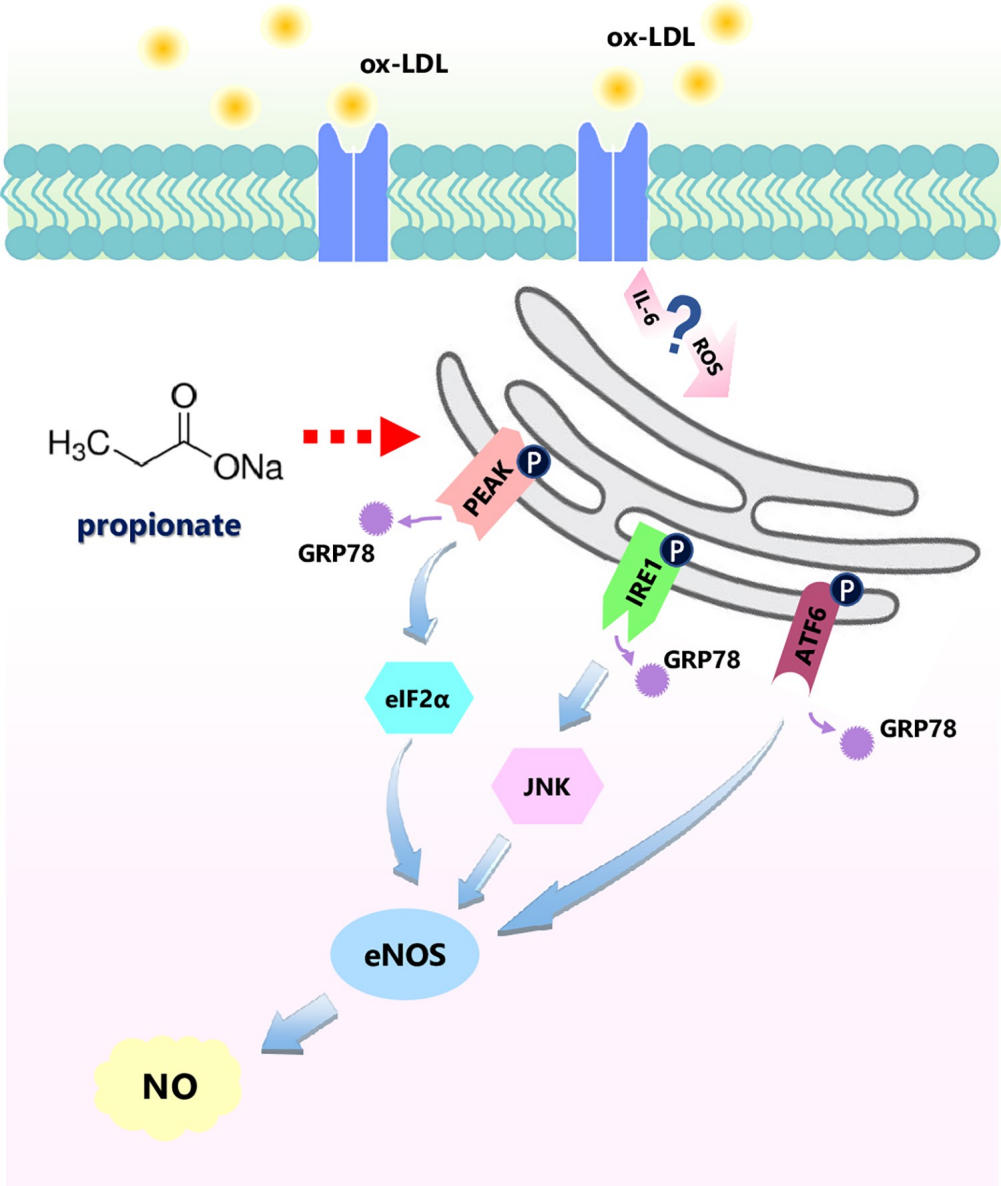
Proposed mechanism of propionate-mediated prevention of ox-LDL-induced downregulation of eNOS phosphorylation and NO production in HCMECs. Ox-LDL upregulates the expression of GRP78, thereby activating the UPR pathway. Activating the PERK/eIF2a, IRE1/JNK and ATF6 pathways leads to downregulating eNOS phosphorylation and NO production in HCMECs. Propionate upregulates the ox-LDL-induced decrease of eNOS phosphorylation and NO production, possibly mediated by inhibiting the UPR pathway.

This study explored the impact of lipid metabolism on coronary microcirculation. As shown in Fig 6, we demonstrated that ox-LDL damages the function of coronary microvascular endothelial cells and reduces NO production, relating to the activation of ER stress, inflammation, and oxidative stress. This finding provides evidence for the timing of dyslipidemia clinical intervention, blood lipid regulation should be earlier than epicardial coronary atherosclerosis. Further research confirmed that propionate can reduce the adverse effects of ox-LDL and protect coronary microcirculation function; the mechanism is inhibition of ER stress, perhaps involving inflammation and oxidative stress. These results provide new ideas for treating coronary microcirculation dysfunction in clinical practice. However, this study has yet to explore the receptor and signal in ox-LDL and ER stress. We speculate that inflammatory factors and ROS may mediate the above process, and more molecular signals need further clarification. In addition, we only observed the effect of propionate on ox-LDL. Research about acetate and butyrate needs to be conducted to facilitate the clinical application potential of SCFA.

## Materials & methods

### Reagents

ox-LDL was obtained from Guangzhou Zhongshan University School of Public Health, Guangzhou, China. Propionate was purchased from Sigma-Aldrich, US. ER stress antibodies were acquired from Sigma-Aldrich and Abcam, UK (the catalog number of reagents, antibodies, siRNA, and kits is shown in S1 Table in S1 File). Only molecular biology- or cell culture-grade reagents were used.

### Cell culture

Human Cardiac Microvascular Endothelial Cell (HCMEC) and Endothelial Cell Medium 1001 were purchased from ScienCell Research Laboratories, USA. Cells were cultured at 37°C under 5% CO_2_ and grown in ECM 1001 supplemented with 5% fetal bovine serum (FBS), 50 U/ml penicillin, and 50 μg/ml streptomycin. The cells grew to 70–80% confluent (logarithmic growth phase) and were treated with drugs. To assess the effect of ox-LDL on HCMECs, cells were incubated with different concentrations (50–150 μg/ml) ox-LDL or 100 μg/ml ox-LDL for 12–48 hours at 37°C under 5% CO_2_. To explore the role of UPR, cells were transfected with Stealth Select RNAi or pretreated with UPR inhibitor drugs for 6 hours, then exposed to ox-LDL (100μg/ml) for 24 hours at 37°C under 5% CO_2_. To clarify the protective effect of propionate, HCMECs were treated with different concentrations (10 and 20 mM) of propionate for 2 hours and then exposed to ox-LDL (100μg/ml) for 24 hours at 37°C under 5% CO_2_.

### Transfection

To knock down the expression of target genes, HCMECs were transfected with the transfection mix containing Lipofectamine RNAiMAX transfection reagent (Thermo Fisher Scientifc, US) and small interference RNAs (siRNAs): 10 nM Stealth Select RNAi (Thermo Fisher Scientifc, US) directed against protein kinase R-like ER kinase (PERK) (HSS190343), IRE1 (HSS140847) or activating transcription factor 6 (ATF6) (HSS117915). Stealth RNAi Negative Control Duplex (Low GC) was used as a negative control. After adding the Stealth Select RNAi to the cells, incubate the cells for 48 hours at 37°C in a CO_2_ incubator. The culture medium was replaced with fresh ECM 1001 supplemented with 5% FBS at 12 h after transfection. The expression levels of silenced genes were evaluated by western blot analysis (BioRad, Canada) and real-time PCR (Applied Biosystems, US).

### Western blot analysis

HCMECs were collected and lysed with RIPA lysis buffer [50mM Tris (pH 7.4), 150mM NaCl, 1% Triton X-100, 1% sodium deoxycholate, 0.1% SDS] and protease and phosphatase inhibitor (Beyotime, China) after treatment. The total protein concentration was determined using the bicinchoninic acid (BCA) reagent (Beyotime, China). Protein samples (50–100 μg each) were loaded and separated by 10% SDS-polyacrylamide gel electrophoresis (SDS-PAGE). After gel electrophoresis, proteins were transferred to a polyvinylidene fluoride membrane. The membranes were trimmed according to the protein sample width, and all proteins were strictly retained during the modification process. After blocking in 5% nonfat milk, the membranes were probed with primary antibodies for GRP78 (1:1000), p-eNOS^Ser1177^ (1:1000), eNOS (1:1000), PERK (1:500), p-eIF2α (1:1000), eIF2α (1:500), IRE-1 (1:1000), p-JNK (1:500), JNK (1:1000), ATF6 (1:1000), or β-actin (1:5000) and subsequently labeled with horseradish peroxidase-conjugated secondary antibody. The bands were visualized by the enhanced chemiluminescence reagent (Millipore, US) and autoradiography, and protein gray analysis was proceeded with Quantity One (BioRad, Canada). All results were representative of three independent experiments.

### Immunofluorescence

Aspirate cell medium after treatment, cover cells with 4% formaldehyde in PBS for 15 minutes at room temperature. Aspirate fixative, rinse three times with PBS for 5 minutes each. Cells were permeabilized for 10 minutes in 0.1% Triton X-100 before blocking in 3% BSA for 30 minutes at room temperature. After incubation with the anti-ATF6 (1:200) primary antibody, incubate overnight at 4°C. Rinse three times with PBS for 5 minutes each. Incubate specimen in Dylight Fluor secondary antibody (EarthOx, US) diluted in phosphate-buffered saline for 2 hours at room temperature in dark. Rinse with PBS. The stained cells were examined under a fluorescence microscope (Olympus, Japan).

### Determination of NO generation

Following the various treatments, ECM 1001 of HCMECs was collected to determine NO levels by a colorimetric assay kit involving the Griess reaction (Beyotime, China). 50 μl of supernatants were collected and mixed with equal volumes of Griess Reagent Ⅰ (50 μl) and Griess Reagent Ⅱ (50 μl) for 10 minutes at room temperature. Absorbance was read at 540 nm. NaNO_2_ (0, 1, 2, 5, 10, 20, 40, 60, 100 μm) was diluted by ECM 1001 with 5% FBS to generate a standard curve. The NO production in the cultured medium was estimated by the NO_2_ - concentration. The detailed procedures were performed according to the manufacturer’s instructions.

### Real-time quantitative PCR

Total RNA was extracted using RNAiso Plus reagent (Takara, Japan), and 2 μg of RNA from each sample was reversely transcribed into cDNA using PrimeScript 1st Strand cDNA Synthesis Kit (Takara). The cDNA (5 μL) was amplified using TB Green Advantage qPCR premix (Takara) and primers. The primers used for amplification are listed in S2 Table in S1 File. Amplification was carried out starting with an initial step at 95°C for 45 seconds, followed by 40 cycles of amplification (95°C for 5 seconds and 60°C for 31 seconds) by using an ABI 7500 real-time PCR system (Applied Biosystems, USA). GAPDH was used as an internal control, and data were expressed as the ratio of target mRNA to GAPDH mRNA. All results were representative of three independent experiments.

### Statistical analysis

Data were expressed as means ± standard deviation (SD). Multiple comparisons were conducted using analysis of variance (ANOVA), followed by the least-significant difference (LSD) test. P<0.05 was recognized as statistically significant.

## Supporting information

S1 Fig The effects of ox-LDL and propionate on HCMECs apoptosis. (A) The apoptosis levels of HCMECs were detected by Annexin V-FITC apoptosis analysis after ox-LDL treatment at various concentrations (0, 50, 100, or 150 μg/ml) for 24 hours. (B) The apoptosis levels of HCMECs were detected after exposure to 100 μg/ml ox-LDL at various times (0, 12, 24, or 48 hours). (C) The apoptosis levels of HCMECs were detected after exposure to ox-LDL (100 μg/ml, 24 hours) or/and propionate (10 and 20 mM). The data were expressed as the mean ± SD, n = 3. Compared with the control, *P<0.05; **P<0.01. Compared with ox-LDL, #P<0.05; ##P<0.01.
(TIF)

S2 Fig The impact of propionate on ox-LDL-induced inflammatory and oxidative stress in HCMECs. Cells were treated with different concentrations (10 and 20 mM) of propionate for 2 hours and then exposed to ox-LDL (100μg/ml, 24 hours). (A) ELISA detected the concentration of IL-1β and IL-6. (B) ELISA detected the concentration of TNF-α. (C) ROS production in HCMECs. The data were expressed as the mean ± SD, n = 3. Compared with the control, *P<0.05; **P<0.01. Compared with ox-LDL, #P<0.05; ##P<0.01.
(TIF)

S1 Raw images (PDF)

S1 File (DOCX)
