# Supplementary figures and images for: The short-chain fatty acid propionate prevents ox-LDL-induced coronary microvascular dysfunction by alleviating endoplasmic reticulum stress in HCMECs
Source: PLoS One. 2024 May 30;19(5):e0304551. doi: 10.1371/journal.pone.0304551 (PMC11139260; doi:10.1371/journal.pone.0304551)

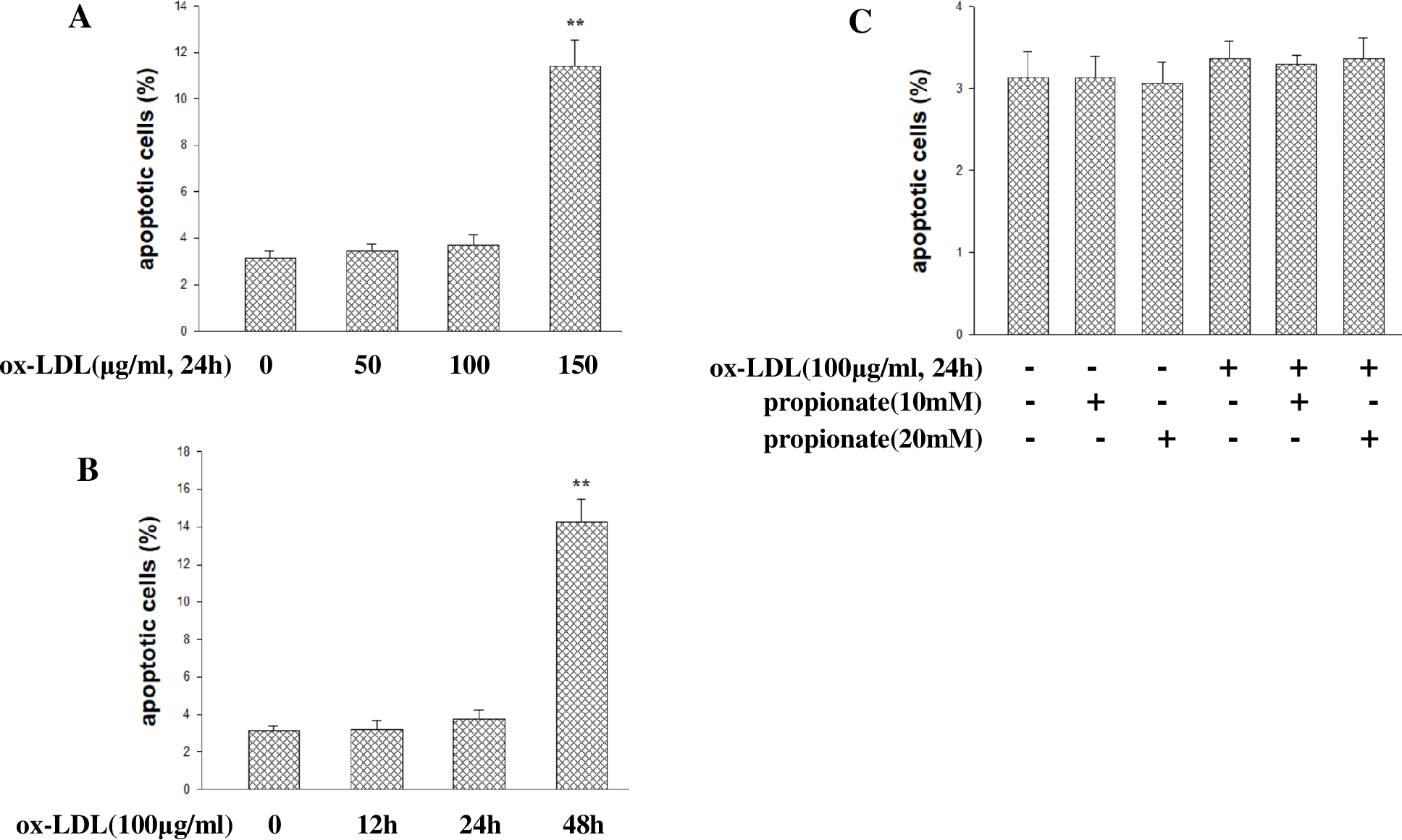

Supplement: S1 Fig — (A) The apoptosis levels of HCMECs were detected by Annexin V-FITC apoptosis analysis after ox-LDL treatment at various concentrations (0, 50, 100, or 150 μg/ml) for 24 hours. (B) The apoptosis levels of HCMECs were detected after exposure to 100 μg/ml ox-LDL at various times (0, 12, 24, or 48 hours). (C) The apoptosis levels of HCMECs were detected after exposure to ox-LDL (100 μg/ml, 24 hours) or/and propionate (10 and 20 mM). The data were expressed as the mean ± SD, n = 3. Compared with the control, *P<0.05; **P<0.01. Compared with ox-LDL, #P<0.05; ##P<0.01. (TIF) [file pone.0304551.s001.tif]

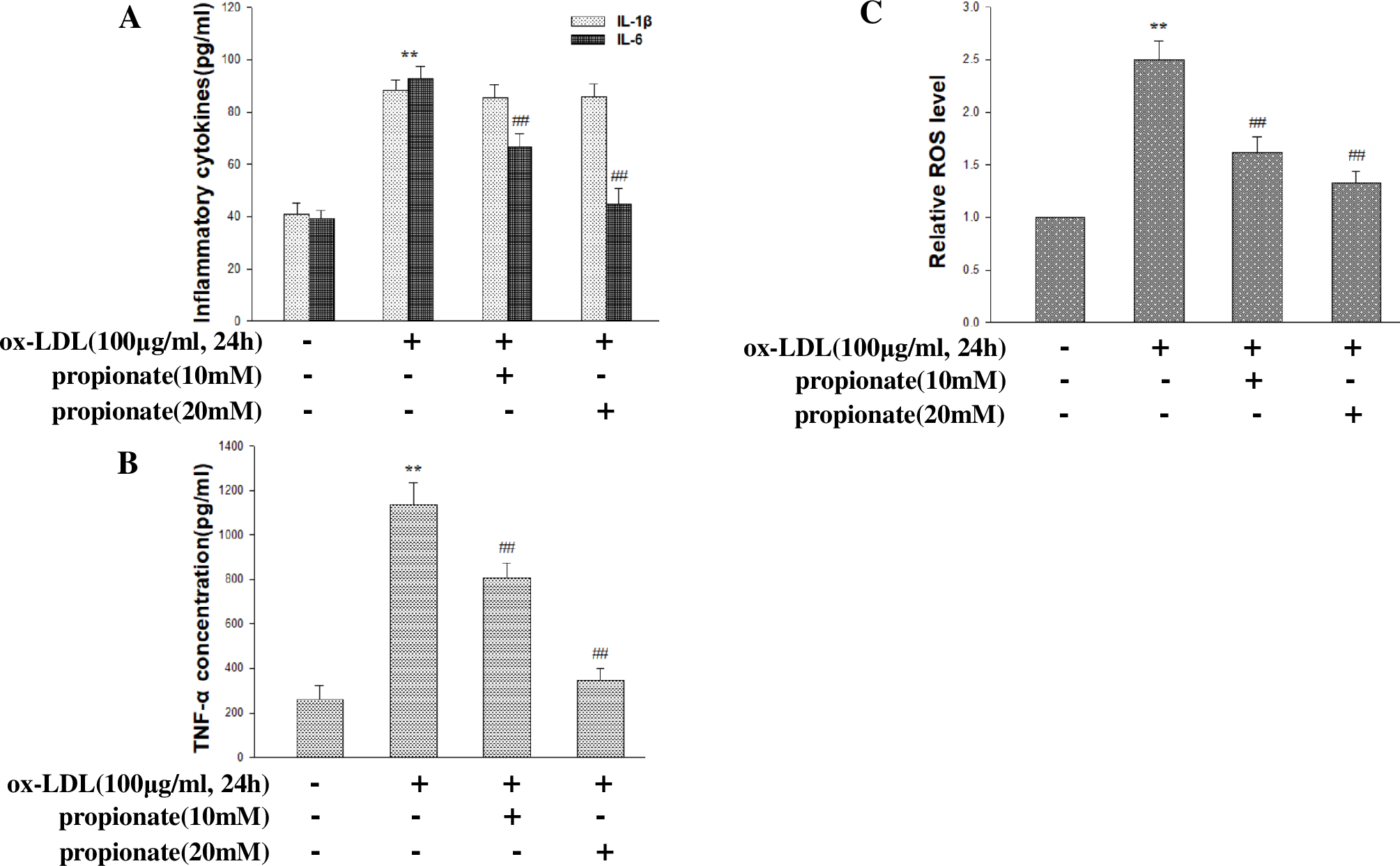

Supplement: S2 Fig — Cells were treated with different concentrations (10 and 20 mM) of propionate for 2 hours and then exposed to ox-LDL (100μg/ml, 24 hours). (A) ELISA detected the concentration of IL-1β and IL-6. (B) ELISA detected the concentration of TNF-α. (C) ROS production in HCMECs. The data were expressed as the mean ± SD, n = 3. Compared with the control, *P<0.05; **P<0.01. Compared with ox-LDL, #P<0.05; ##P<0.01. (TIF) [file pone.0304551.s002.tif]
