# Supplementary material for: The short-chain fatty acid propionate prevents ox-LDL-induced coronary microvascular dysfunction by alleviating endoplasmic reticulum stress in HCMECs
Source: PLoS One. 2024 May 30;19(5):e0304551. doi: 10.1371/journal.pone.0304551 (PMC11139260; doi:10.1371/journal.pone.0304551)
Supplement: S1 Raw images — (PDF) [file pone.0304551.s003.pdf]

Western blot original picture For

The short-chain fatty acid propionate prevents ox-LDL-induced coronary microvascular dysfunction by alleviating endoplasmic reticulum stress in HCMECs

Dan Hong, Wen Tang, Fei Li, Yating Liu, Xiao Fu, Qin Xu

Figure 1A

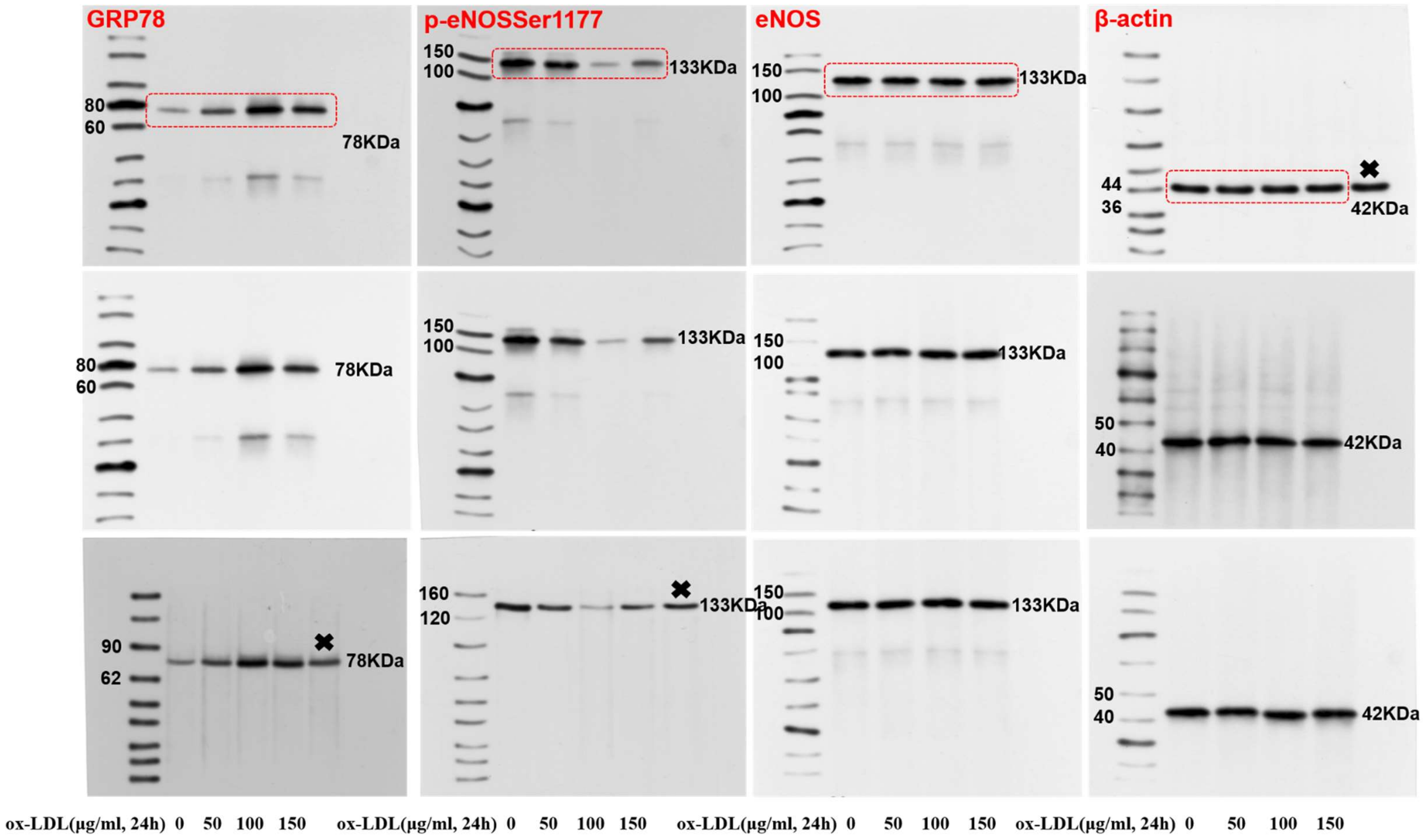

Figure 1B

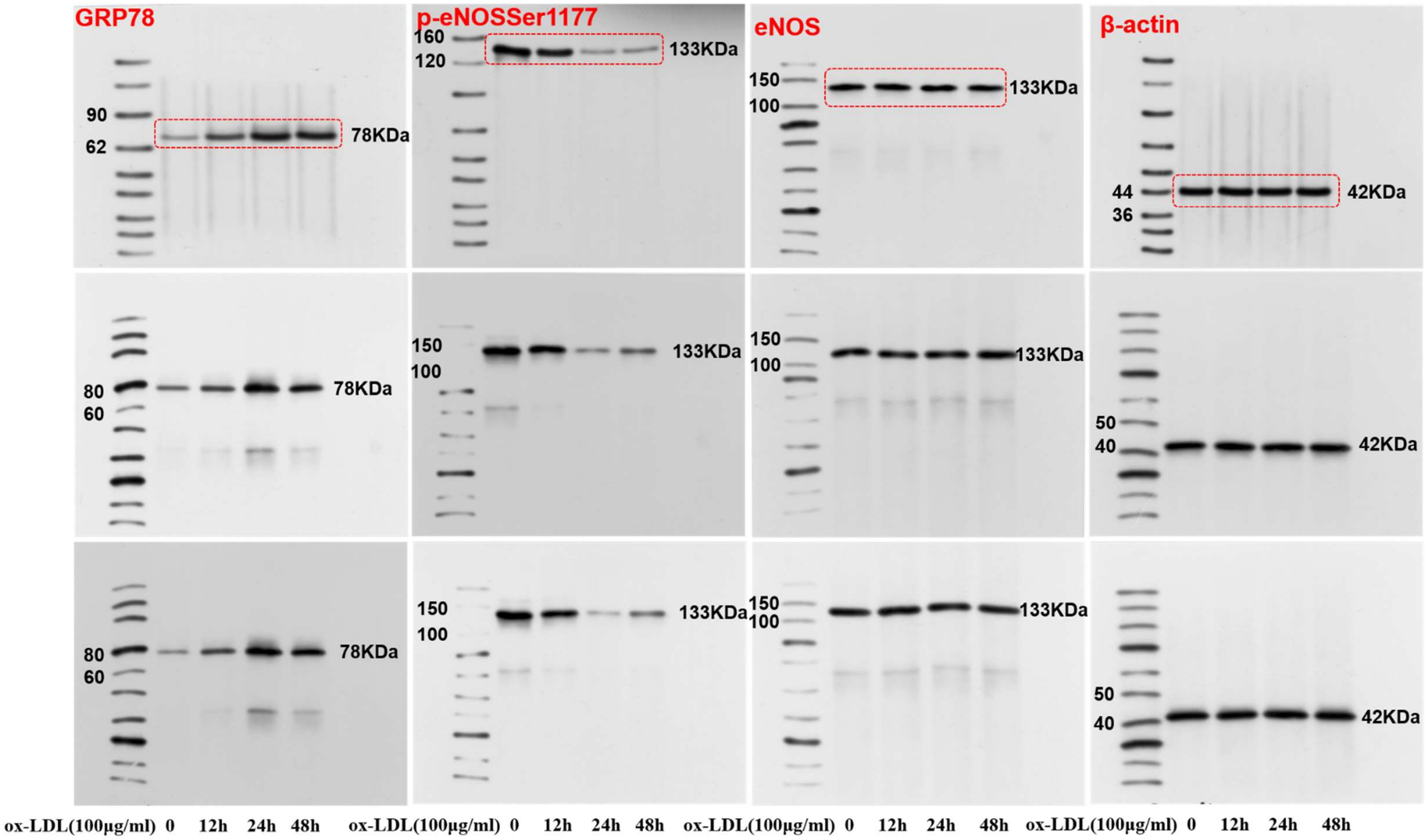

GRP78
p-eNOSSer1177
eNOS
β-actin
90
62
78KDa
160
120
133KDa
150
100
133KDa
44
36
42KDa
80
60
78KDa
150
100
133KDa
150
100
133KDa
50
40
42KDa
80
60
78KDa
150
100
133KDa
150
100
133KDa
50
40
42KDa
ox-LDL(100μg/ml) 0 12h 24h 48h
ox-LDL(100μg/ml) 0 12h 24h 48h
ox-LDL(100μg/ml) 0 12h 24h 48h
ox-LDL(100μg/ml) 0 12h 24h 48h

Figure 2A

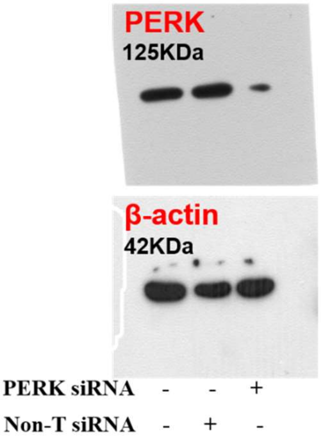

Figure 2C

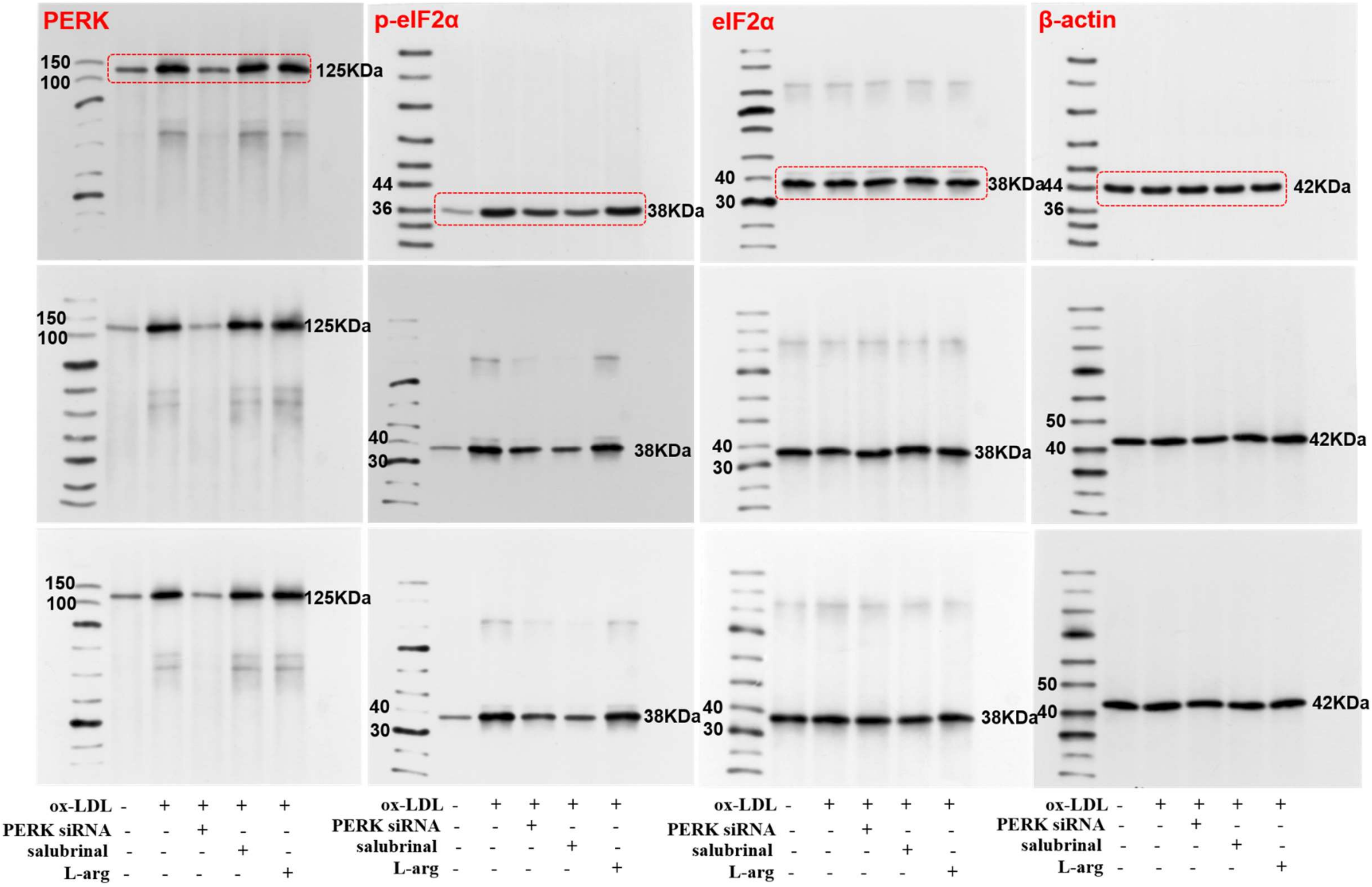

Figure 2D

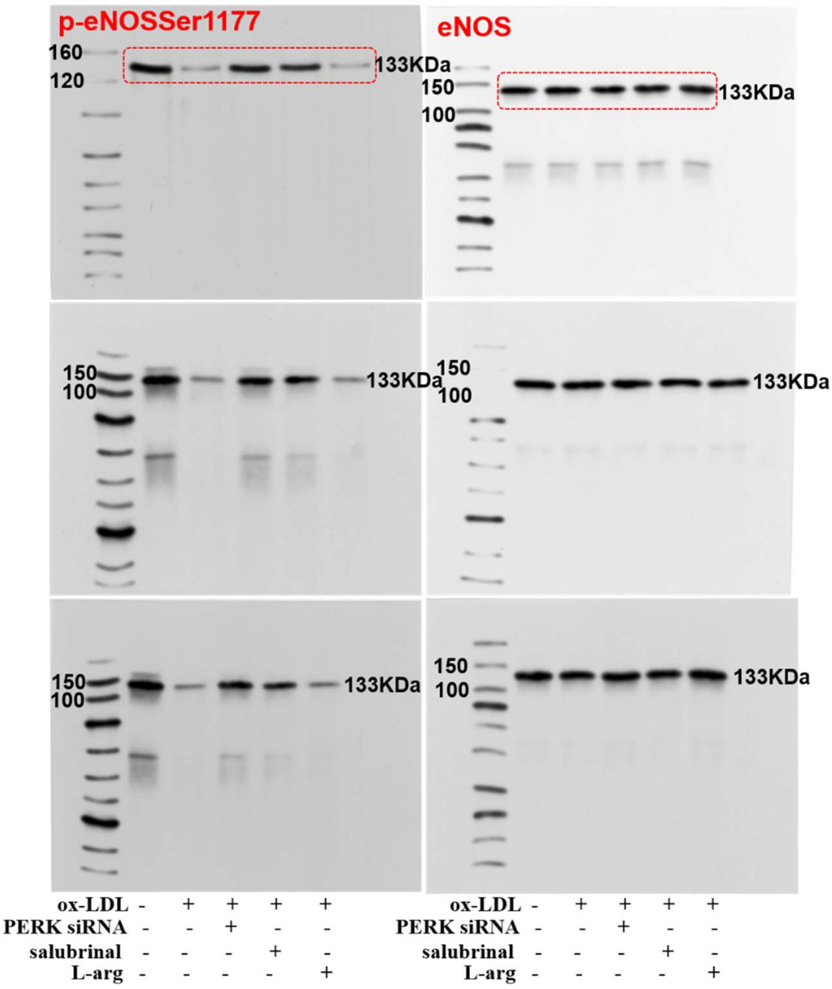
p-eNOSSer1177
eNOS
160
120
150
100
133KDa
ox-LDL - + + + +
PERK siRNA - - + - -
salubrinal - - - + -
L-arg - - - - +

Figure 3A

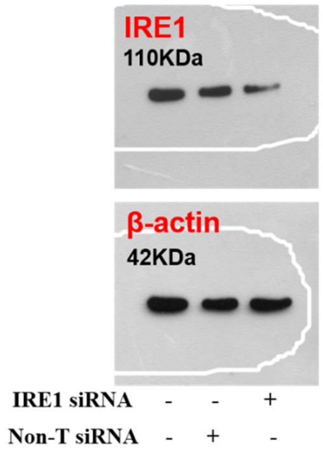

Figure 3C

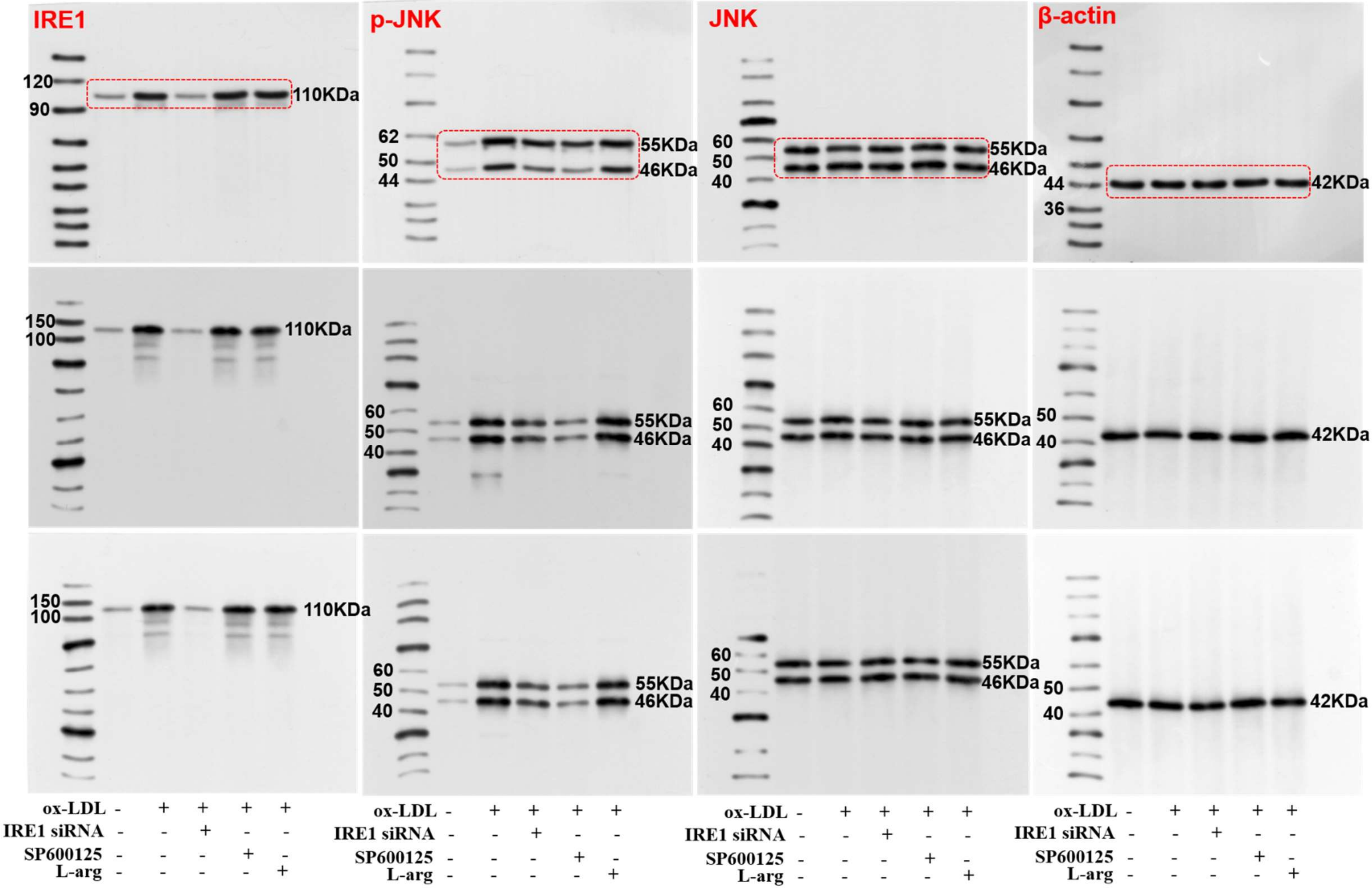

Figure 3D

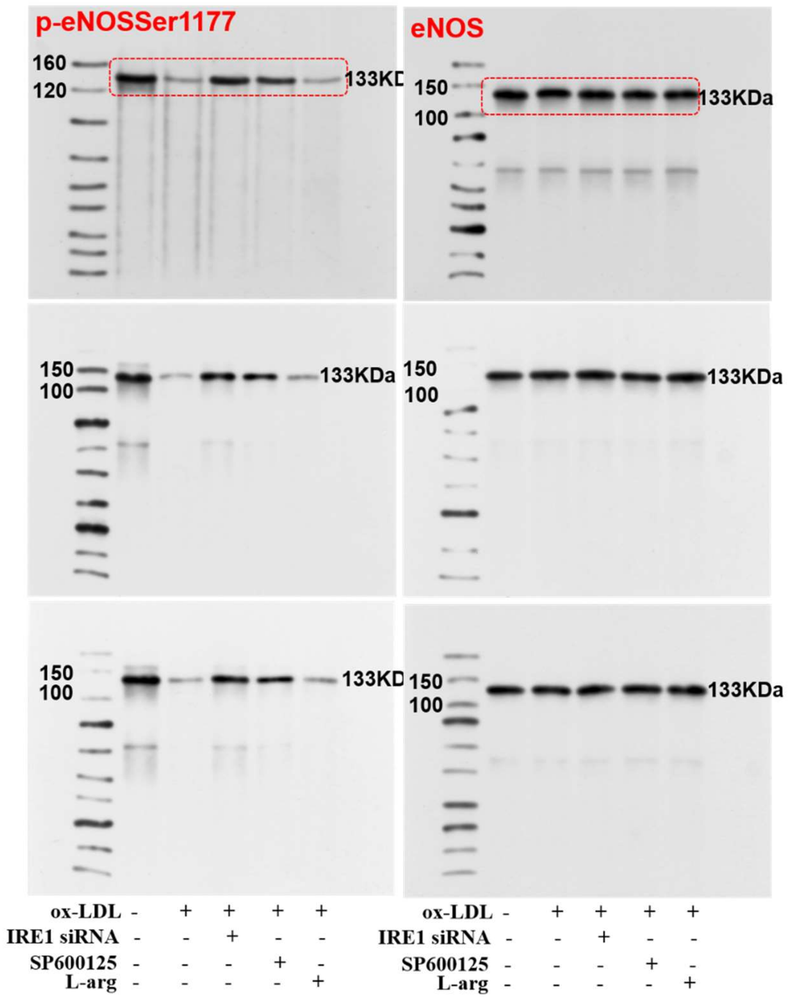
p-eNOSSer1177
160
120
133KD
eNOS
150
100
133KDa
150
100
133KDa
150
100
133KDa
150
100
133KD
150
100
133KDa
ox-LDL - + + + +
IRE1 siRNA - - + - -
SP600125 - - - + -
L-arg - - - - +
ox-LDL - + + + +
IRE1 siRNA - - + - -
SP600125 - - - + -
L-arg - - - - +

Figure 4A

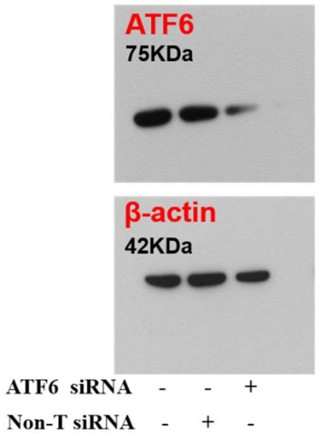

Figure 4D

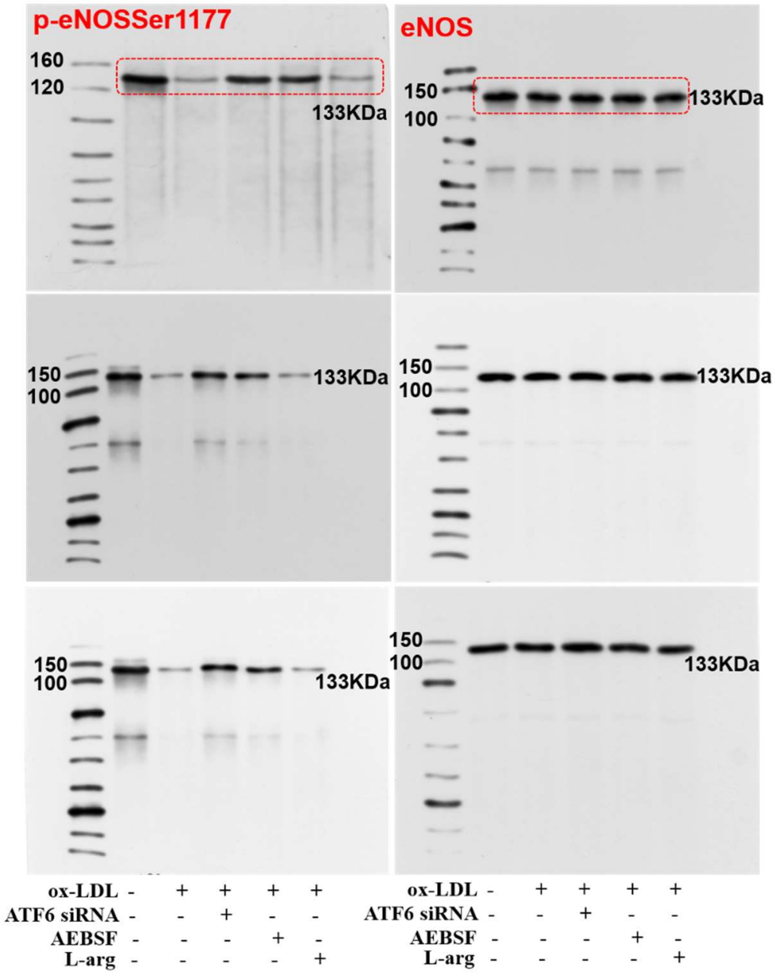

Figure 5A

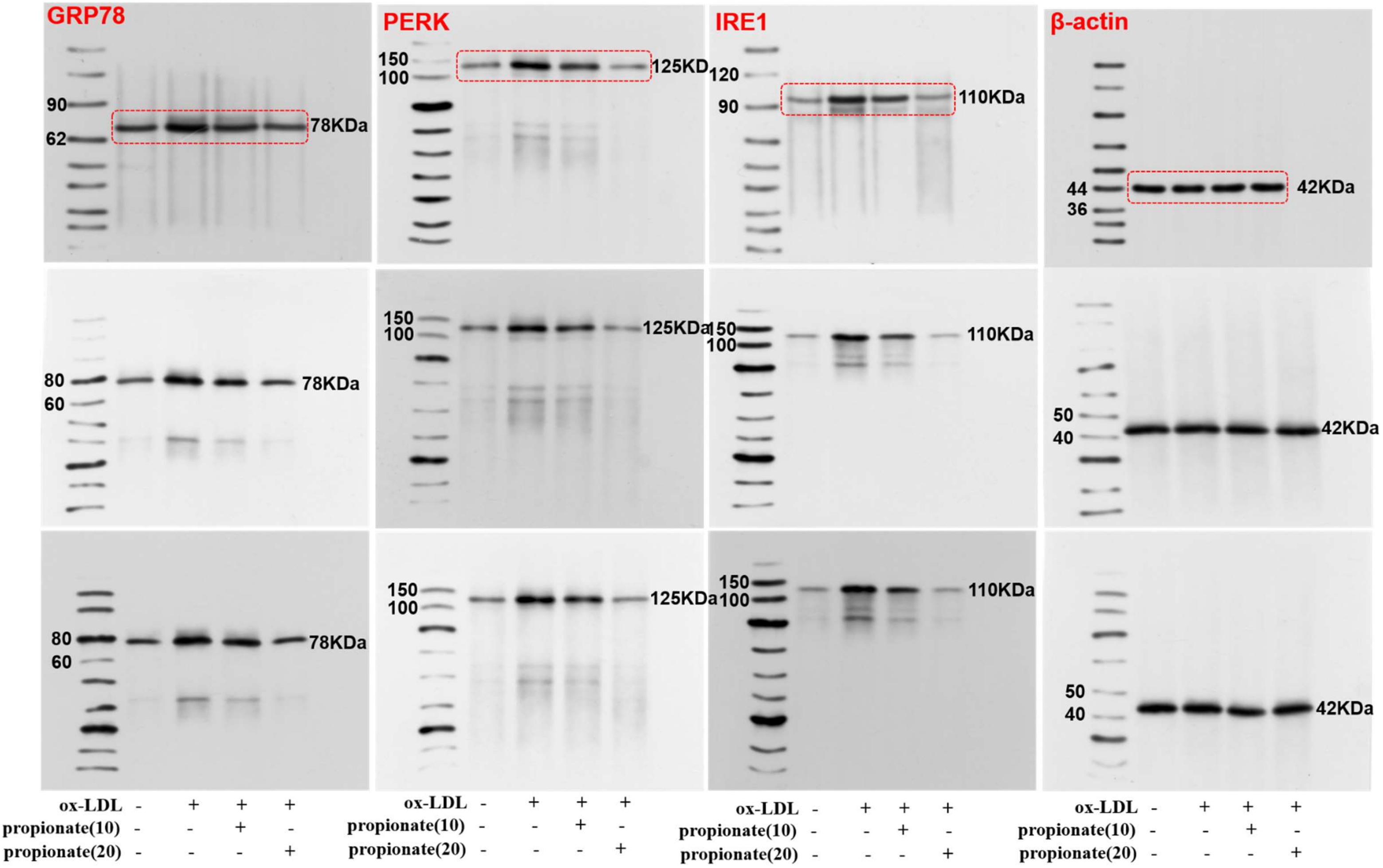
GRP78
PERK
IRE1
β-actin
78KDa
125KD
125KDa
110KDa
42KDa
90
62
80
60
150
100
120
50
44
40
36
ox-LDL - + + +
propionate(10) - - + -
propionate(20) - - - +

Figure 5C

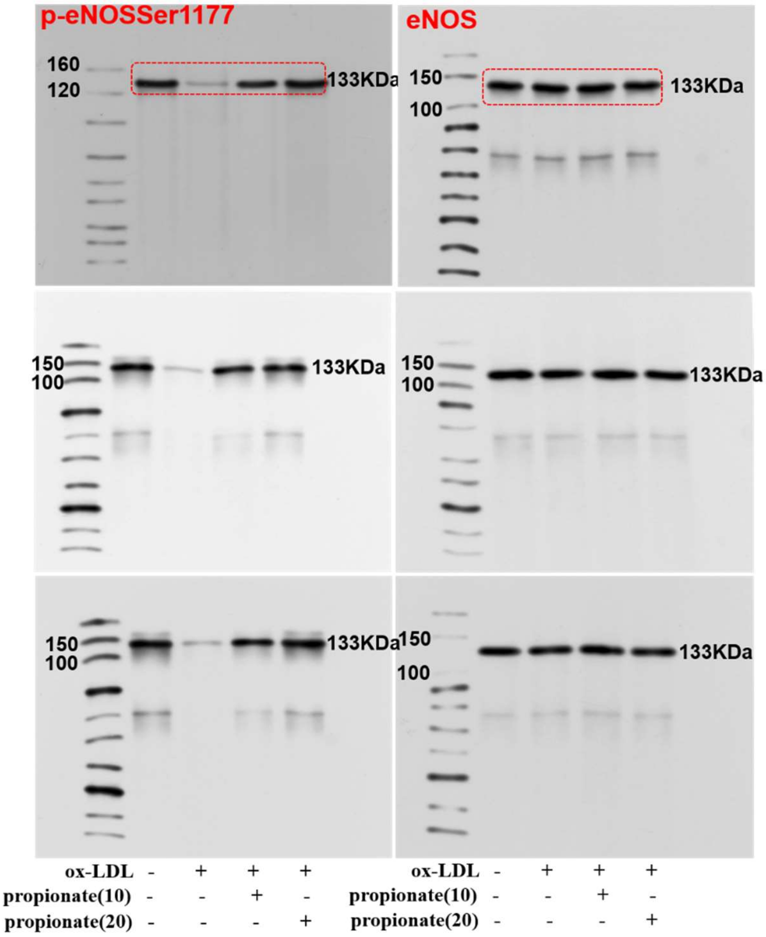
p-eNOSSer1177
eNOS
160
120
133KDa
150
100
133KDa
150
100
133KDa
150
100
133KDa
150
100
133KDa
150
100
133KDa
ox-LDL - + + +
propionate(10) - - + -
propionate(20) - - - +
ox-LDL - + + +
propionate(10) - - + -
propionate(20) - - - +
