# Supplementary material for: The short-chain fatty acid propionate prevents ox-LDL-induced coronary microvascular dysfunction by alleviating endoplasmic reticulum stress in HCMECs
Source: PLoS One. 2024 May 30;19(5):e0304551. doi: 10.1371/journal.pone.0304551 (PMC11139260; doi:10.1371/journal.pone.0304551)
Supplement: S1 File — (DOCX) [file pone.0304551.s004.docx]

**Supplementary For**

**The short-chain fatty acid propionate prevents ox-LDL-induced coronary microvascular dysfunction by alleviating endoplasmic reticulum stress in HCMECs**

**Dan Hong, Wen Tang, Fei Li, Yating Liu, Xiao Fu, Qin Xu**

**Table S1.** The catalog of reagents, antibodies, siRNA, and kits.

| **Reagent type** | **Designation** | **Source** | **Catalog** |
| --- | --- | --- | --- |
| Cell line  (*Homo sapiens*) | Human Cardiac Microvascular Endothelial Cell **(**HCMEC**)** | ScienCell | 6000 |
|  | Endothelial Cell Medium | ScienCell | 1001 |
| Biochemical Reagent | ox-LDL | Zhongshan University School | YB-002 |
|  | Propionate | Sigma-Aldrich | P1880 |
|  | salubrinal | Sigma-Aldrich | 324895 |
|  | SP600125 | Sigma-Aldrich | 420119 |
|  | AEBSF | Sigma-Aldrich | 101500 |
|  | L-arg | Sigma-Aldrich | A8094 |
| Antibody | anti-GRP78 | Abcam | ab108615 |
|  | anti-eNOS(p-Ser1177) | Abcam | ab215717 |
|  | anti-eNOS | Abcam | ab252439 |
|  | anti- PERK | Abcam | ab79483 |
|  | anti- p-eIF2α | Sigma-Aldrich | 07-760-I |
|  | anti- eIF2α | Santa Cruz | SC-133132 |
|  | anti- IRE1 | Abcam | ab235171 |
|  | anti- p-JNK | Sigma-Aldrich | J4750 |
|  | anti- JNK | Sigma-Aldrich | SAB4200176 |
|  | anti- ATF6 | Abcam | ab37149 |
|  | anti-β-actin | Abcam | ab8226 |
|  | Goat Anti-Rabbit IgG H&L (HRP) | Abcam | ab97051 |
|  | Goat Anti-Mouse IgG HRP Conjugate (H+L) | Sigma-Aldrich | 71045-M |
|  | Primary Antibody Dilution Buffer | Shanghai Beyotime | P0023A |
| siRNA | PERK Stealth RNAi | Thermo Fisher Scientifc | HSS190343 |
|  | IRE1 Stealth RNAi | Thermo Fisher Scientifc | HSS140847 |
|  | ATF6 Stealth RNAi | Thermo Fisher Scientifc | HSS117915 |
| Kit | RIPA lysis buffer | Shanghai Beyotime | P0013K |
|  | Protease and Phosphatase Inhibitor Cocktail Kit | Shanghai Beyotime | P1046 |
|  | Enhanced BCA Protein Assay Kit | Shanghai Beyotime | P0010S |
|  | RNAiso Plus reagent | Takara Bio | 9108 |
|  | PrimeScript 1st Strand cDNA Synthesis Kit | Takara Bio | 6110A |
|  | TB Green Advantage qPCR premix | Takara Bio | 639676 |
|  | Nitric Oxide (NO) Assay Kit | Shanghai Beyotime | S0021S |
|  | Annexin V-FITC Apoptosis Kit | Shanghai Beyotime | C1062M |
|  | Interleukin -1β(IL-1β) Assay Kit | Nanjing Jiancheng | H002-1-2 |
|  | Interleukin -6(IL-6) Assay Kit | Nanjing Jiancheng | H007-1-2 |
|  | Tumor Necrosis Factor-α (TNF-α) Assay Kit | Nanjing Jiancheng | H052-1-2 |
|  | Reactive Oxygen Species (ROS) Assay Kit | Shanghai Beyotime | S0033S |

**Table S2.** Primers for Real-time quantitative PCR.

| **Oligonucleotide target gene**  **(F, forward and R, reverse)** | **Sequence (5`- 3`)** |
| --- | --- |
| PERKF | TTGTCGCCAATGGGATAG |
| PERKR | CAGTCAGCAACCGAAACC |
| IRE1F | CCAGTACATTGCCATCGAGC |
| IRE1R | TCCAGGGATTCCTTTTCTATT |
| ATF6F | CCATTGCTTTACATTCCTCCAC |
| ATF6R | CAGGGTCCCACGCTCAGT |
| GAPDHF | CTGCACCACC AACTGCTTAG |
| GAPDHR | AGGTCCACCACTGACACGTT |

**Results：**

**The effects of ox LDL and propionate on the level of**HCMECs**apoptosis**

HCMECs were treated with varying concentrations of ox-LDL and propionate to understand the effects on HCMEC apoptosis. Annexin V-FITC apoptosis analysis showed that treatment with ox-LDL (50-100 μg/ml) and 12-24 hours does not affect cell apoptosis rate (Supplementary Fig. S1A-B). Moreover, propionate (10-20 mM) treatment does not cause cell apoptosis (Supplementary Fig. S2C). However, after high concentration (150 μg/ml) or prolonged (48 hours) treatment with ox-LDL, the apoptosis rate of cells increases (Supplementary Fig. S1A-B). These findings suggest that high concentrations or prolonged exposure to ox-LDL can induce cell apoptosis.


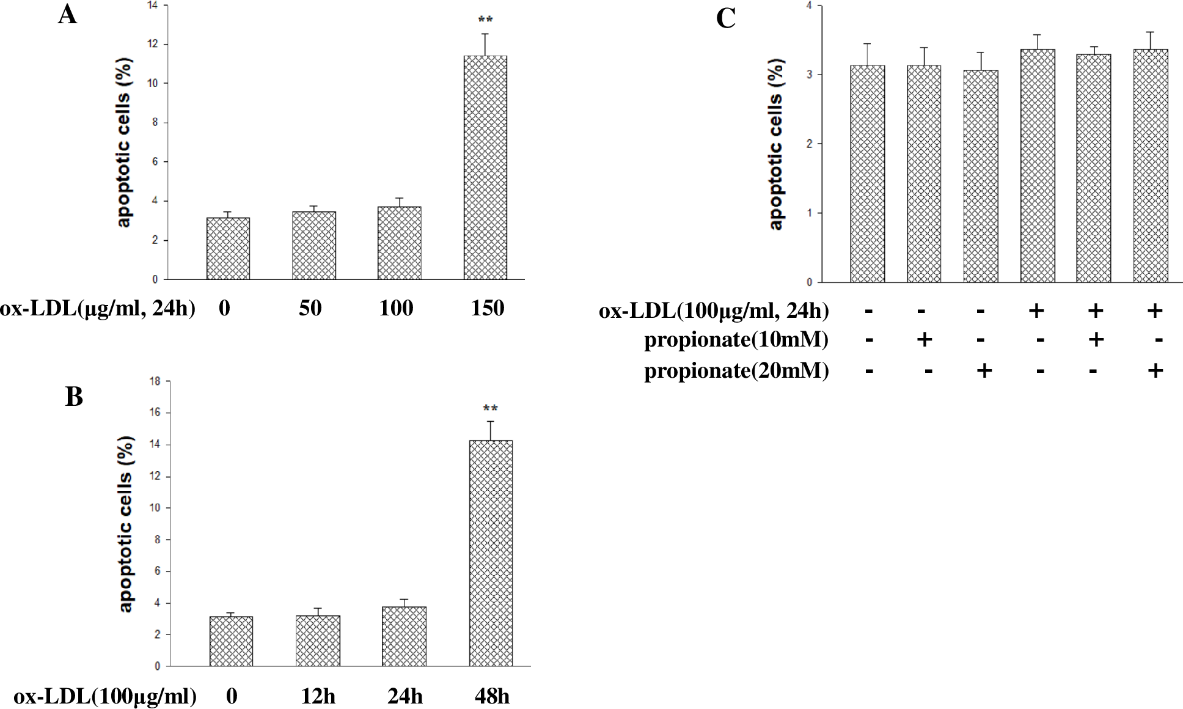


**Supplementary Figure S1**. The effects of ox LDL and propionate on the level of HCMECs apoptosis.

**Effects of propionate on ox-LDL-induced inflammation and oxidative stress in HCMECs**

Inflammatory and oxidative stress are vital in the pathogenesis of coronary microcirculation dysfunction. In order to explore the effect of propionate on ox-LDL-induced inflammation and oxidative stress, HCMECs were exposed to ox-LDL (100 μg/ml) for 24 hours. As expected, the level of inflammatory cytokines, including interleukin (IL)-1β, IL-6, and tumor necrosis factor (TNF)-α were elevated (Supplementary Fig. S2A-B). Before ox-LDL treatment, pretreatment of HCMECs with propionate (10-20 mM) reduced the concentration of IL-1β, IL-6, and TNF-α (Supplementary Fig. S2A-B). Furthermore, ox-LDL (100 μg/ml, 24 hour) treatment triggered the increase in reactive oxygen species (ROS) (Supplementary Fig. S2C), propionate (10-20 mM) reduced the level of ROS resulting from ox-LDL treatment (Supplementary Fig. S2C). Higher-dose propionate shows a more substantial protective effect. These findings indicate that propionate reduced the inflammatory and oxidative stress in ox-LDL-treated HCMECs.


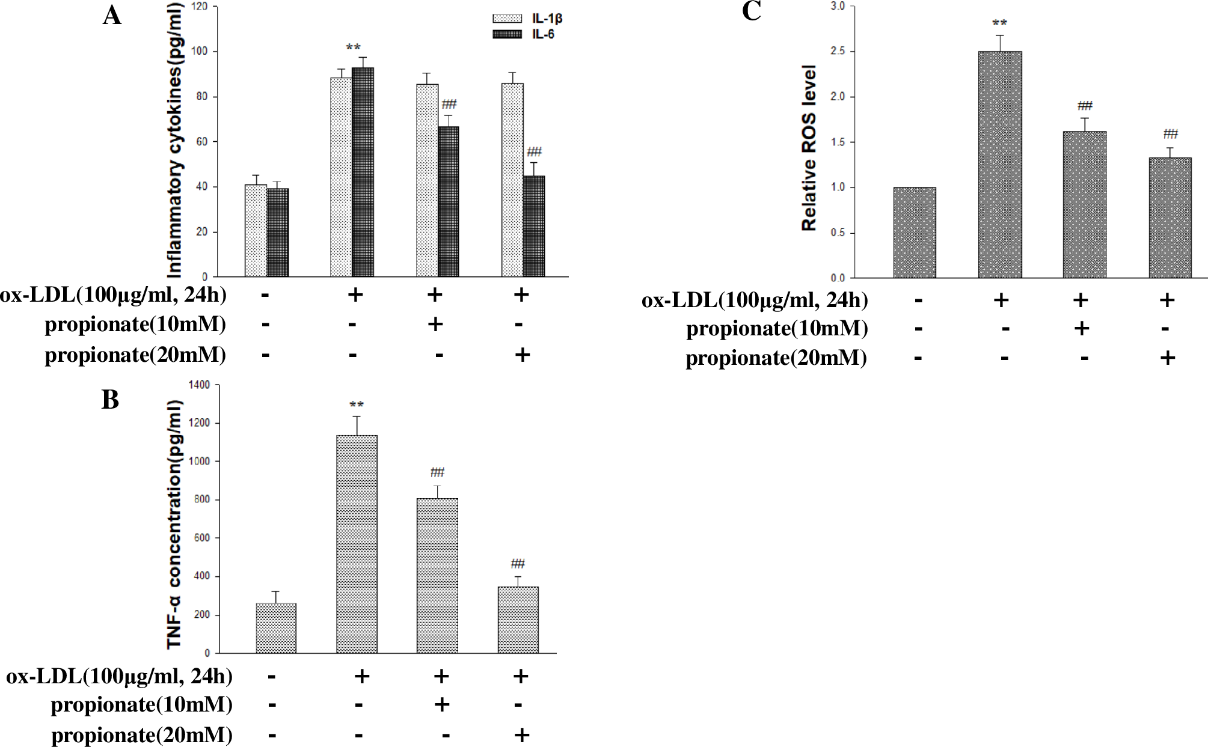


**Supplementary Figure S2**. The impact of propionate on ox-LDL-induced inflammation and oxidative stress in HCMECs.

**Methods**

**Detection of apoptosis**

HCMECs were digested in a 6-well plate, firstly incubated with different concentrations (50-150 μg/ml) ox-LDL or 100 μg/ml ox-LDL for 12-48 hours. secondly, cells were treated to different concentrations (10 and 20 mM) for 2 hours of propionate and then exposed to ox-LDL (100μg/ml) for 24 hours. Cells were centrifuged at 1000 rpm for 5 min, resuspended in binding buffer, and labeled with Annexin V and PI probes (Beyotime, China) according to the manufacturer’s instructions and as described in [Ox-LDL induces endothelial cell apoptosis via the LOX-1-dependent endoplasmic reticulum stress pathway. Atherosclerosis. 235(2), 310-317]. Cells were then analyzed using a FACS flow cytometer (Becton Dickinson, NJ). The percentage of Annexin V positive (and propidium iodide positive or negative) cells was determined and compared between the different conditions.

**Inflammatory Cytokines Estimation (IL-1β, IL-6, and TNF-α) by ELISA**

HCMECs were digested in a 6-well plate, treated to different concentrations (10 and 20 mM) for 2 hours of propionate, and then exposed to ox-LDL (100μg/ml) for 24 hours. The supernatant of the HCMECs can be centrifuged (500×g, 5 minutes); 100μl/well supernatant was added to a 96-well ELISA plate and incubated at room temperature for 120 minutes. Wash the plate three times. 100μl/well Biotinylated antibody was added to the ELISA plate and incubated at room temperature for 60 minutes. Wash the board three times. 100μl/well streptavidin labeled with horseradish peroxidase was added and incubated at room temperature for 20 minutes in the dark. Wash the board three times. Add TMB 100μl/well and incubate at room temperature for 15 minutes in the dark. Add 50μl/well termination solution and measure A450 immediately after mixing. The absorbance value and standard curve calculated the concentration of the sample.

**Determination of ROS**

HCMECs were digested in a 6-well plate, treated to different concentrations (10 and 20 mM) for 2 hours of propionate, and then exposed to ox-LDL (100μg/ml) for 24 hours. Fluorescent probes DCFH-DA (1.5ml/well) was added and incubated at 37℃ for 20 minutes in the dark. Wash the plate three times with a serum-free cell culture medium. To estimate intracellular ROS levels, fluorescence was used for excitation (488 nM) and emission (519 nM) via the confocal microscope (Olympus, Japan).

**Figure legends**

**Supplementary Figure S1. The effects of ox-LDL and propionate on HCMECs apoptosis.**

(A) The apoptosis levels of HCMECs were detected by Annexin V-FITC apoptosis analysis after ox-LDL treatment at various concentrations (0, 50, 100, or 150 μg/ml) for 24 hours. (B) The apoptosis levels of HCMECs were detected after exposure to 100 μg/ml ox-LDL at various times (0, 12, 24, or 48 hours). (C) The apoptosis levels of HCMECs were detected after exposure to ox-LDL (100 μg/ml, 24 hours) or/and propionate (10 and 20 mM). The data were expressed as the mean ± SD, n=3. Compared with the control, *P<0.05; **P<0.01. Compared with ox-LDL, #P<0.05; ##P<0.01.

**Supplementary Figure S2. The impact of propionate on ox-LDL-induced inflammatory and oxidative stress in HCMECs.**

Cells were treated with different concentrations (10 and 20 mM) of propionate for 2 hours and then exposed to ox-LDL (100μg/ml, 24 hours). (A) ELISA detected the concentration of IL-1β and IL-6. (B) ELISA detected the concentration of TNF-α. (C) ROS production in HCMECs. The data were expressed as the mean ± SD, n=3. Compared with the control, *P<0.05; **P<0.01. Compared with ox-LDL, #P<0.05; ##P<0.01.
